# Prevalence of Overweight and Obesity and Their Associated Risk Factors in School-Going Children in North Karnataka City

**DOI:** 10.7759/cureus.68356

**Published:** 2024-09-01

**Authors:** Anjali Pandey, Pooja Todalabagi

**Affiliations:** 1 Community Medicine, Maharshi Vishwamitra Autonomous State Medical College, Ghazipur, IND; 2 Community Medicine, Shri B. M. Patil Medical College, BLDE (Deemed to be University), Vijayapura, IND

**Keywords:** north karnataka, body mass index, obesity, overweight, childhood

## Abstract

Background

Malnutrition presents threats to human health, including undernutrition and overweight. Obesity is becoming a worldwide problem affecting all levels of society. Therefore, it is described as a global epidemic with a more pronounced impact in developing countries undergoing rapid epidemiological transitions. An increase in the prevalence of childhood obesity is associated with potential medical complications of obesity in adulthood. The purpose of the study is to estimate the prevalence of obesity and risk factors associated with obesity in school-going children of north Karnataka city.

Methods

This cross-sectional study was conducted in schools in north Karnataka city on 1330 school-going children 10-17 years of age. The overweight and obesity groups were considered using an updated BMI reference of children from two to 18 years ago, according to the International Obesity Task Force (IOTF), and a structured questionnaire was used to assess the socio-demographic characteristics. Chi-square test was used to find out the association between risk factors and overweight and obesity and p value of less than 0.05 was deemed statistically significant.

Results

The prevalence of obesity in this study was 6.5%. It was found that 64 (22.9%) of the children who were obese or overweight were 14 years of age. Eighty (16.6%) obese children belonged to class 4 socio-economic class and 140 (22.0%) obese children used motorized means of transport. The eating habits of obese children were assessed, and it was found that 151 (19.4%) children had mixed diets, 210 (18.5%) obese children consumed junk food, and 183 (19.3%) obese children drank carbonated drinks.

Conclusion

This study suggest that the prevalence of overweight and obesity varies remarkably with different socio-demographic factors. Prevention of childhood obesity is necessary because it is nearly impossible to get children to lose weight and maintain it. Preventive interventions are needed at the individual level to adopt healthy lifestyle behavior and policy-making levels to tackle the obesity problem in the country.

## Introduction

The world today faces a double burden of malnutrition, which incorporates both undernutrition and overweight. The biggest public health challenge of this century is non-communicable diseases, and childhood obesity is a crucial part of this. Childhood obesity was so far thought to be a problem in developed countries; however, it is increasingly being reported in middle-and low-income countries, particularly urban areas [1]. WHO reported that 390 million children and adolescents were overweight in 2022. The prevalence of overweight (including obesity) has risen dramatically from just 8% in 1990 to 20% in 2022. While just 2% of children and adolescents aged five to 19 were obese in 1990, by 2022, 8% of children and adolescents were living with obesity [2]. 

The prevalence of childhood and adolescent obesity is high and continues to rise in low- and middle-income countries (LMICs), while these regions are struggling with undernutrition [3]. In India, the magnitude of overweight ranges from 10% to 30% among children [4].

In each case, the origin of obesity can be traced back to childhood and adolescence. Obesity in childhood is one among the most serious challenges of the 21st century [5]. It is important to recognize childhood obesity early and manage it, because if untreated, it can result in obesity in adulthood with potential medical complications like hypertension, coronary artery disease, cerebrovascular accidents, diabetes mellitus type 2, dyslipidemia, gall stones, premature joint destruction and many others. Additionally, children and adolescents who are obese have been more likely to contract severe coronavirus disease 2019 (COVID-19) during the COVID-19 pandemic, necessitating hospitalization and mechanical ventilation [6]. 

Childhood obesity also has a deep psychosocial impact and is consistently associated with lower scholastic achievements. In addition, obese children suffer from social bias; prejudice and discrimination on the part, not solely of the general public but also of health professionals and this makes them reluctant to seek medical help. Weight once gained is troublesome to lose, and hence prevention is important. This assumes greater significance with regards to children as compared to adults, because they are more susceptible to the constant bombardment by advertisements for energy-dense food. This study is a useful tool for estimating the prevalence of obesity and important risk factors associated with obesity. Accessing and controlling the growing threat of this impending pandemic is the urgency of the hour.

## Materials and methods

A community based cross-sectional study was done for a period of 18 months in the schools of North Karnataka city. The children of age group between 10 to 17 years were enrolled as study participants of the study. The prevalence of obesity among school-going children, as observed by Hussain et al. [7] in north Karnataka city, was 6.82%, using this as p with the formula, n = [DEFF*Np(1-p)]/ [(d2/Z21-α/2*(N-1)+p*(1-p)]. The sample size estimated was 1207 with a 95% confidence level and with a relative error of 20%. After adding a 10% sample loss, the final sample size was 1328, which was approximated to 1330. Out of 28 total schools present in the city, six schools (three were government schools and three were private schools) were chosen using the systematic random sampling technique. All students from class fifth to 10th standard who belonged to the age group of 10-17 years were included in this study. The students having chronic illness or/and history of hospitalization prior to six months of study, physical deformities (of the spine), musculoskeletal and other gross congenital anomalies and students who were not willing to participate in the study and whose parents did not give consent for their wards were excluded from the study.

Institutional ethical clearance was attained (SNMC/IECHSR/2018-19/A-44/1.1). Permission from the competent authority, principal and teachers of the school, were met and explained about the importance of the study for the students and necessary permission was obtained for conducting the study. Parents were also informed by the principal about the study. Oral consent was obtained from all study subjects, before enrolment in the study.

A pilot study was conducted for the validation of the questionnaire by taking 10% of the sample size, and the necessary changes were made to the questionnaire. The pilot study data were included in the sample. Data was collected using pretested and predesigned proforma, which includes socio-demographic information, eating habits, details of physical activity, and anthropometric measurements from the study participants. The anthropometric parameters height, weight, waist, and hip circumference were taken using standardized equipment (stadiometer, weighing machine, and non-stretchable measuring tape, respectively). Body weight was measured (to the nearest 0.5 kg) with the subject standing motionless on the weighing scale, the feet spread 15 cm apart, and the weight was equally distributed on each leg. Subjects were made to remove their footwear and heavy clothing while their weight was being measured. Height was measured (to the nearest 0.5cm) with the subject in an erect position against a vertical surface, with the head positioned so that the top of the external auditory meatus is level with the inferior margin of the bony orbit.

Information was collected on age, gender, religion, family type, parents' education and occupation, total monthly family income, and socioeconomic status. Additional data included a history of chronic illness, hospitalization in the past six months, endocrine disorders, drug intake, and family history of obesity.

Dietary factors were assessed, including the type of diet and the frequency of fried food, junk food, and carbonated drink consumption over the past week. Physical activity and sedentary behavior were evaluated through questions about participation in outdoor and indoor games, household activities, walking, and other exercises. The time spent watching TV, using the internet and mobile devices, and playing video games was also recorded, along with the frequency of these activities per week. The mode of transport to school was noted.

For statistical analysis, data were entered into Excel (Microsoft, Redmond, WA, USA) and analyzed using SPSS software version 20 (IBM Corp., Armonk, NY, USA). The Chi-square test was employed to determine the association between risk factors and overweight/obesity, with a p-value of less than 0.05 considered statistically significant.

## Results

**Figure 1:**
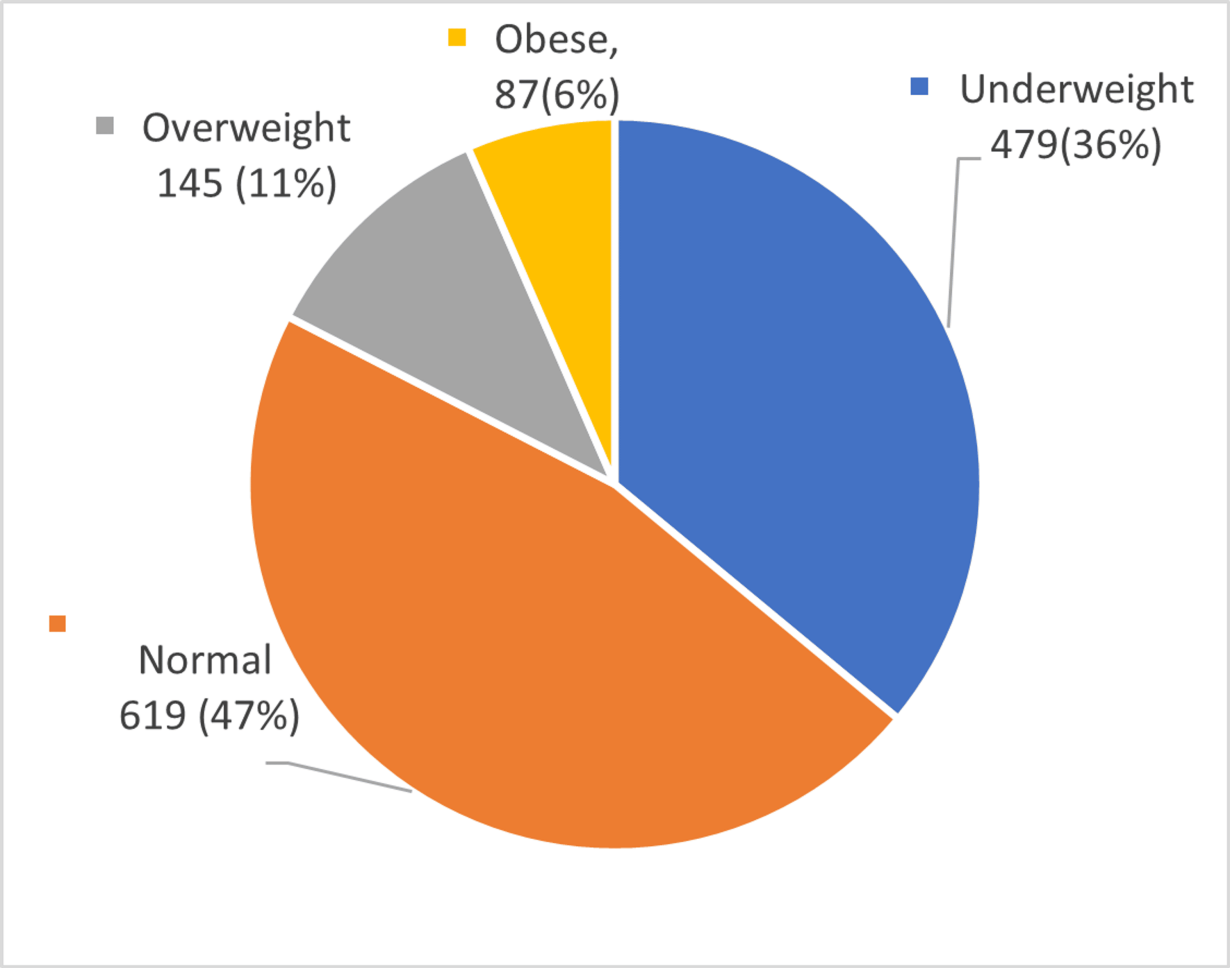
Distribution of study subjects according to BMI

A total of 1330 students were included in the study. Six hundred eighty-five (51.5%) of this study population were boys and 645 (48.5%) were girls. Three hundred fifty-four (26.6%) of the students were aged 15 years and 280 (21.1%) were aged 14 years. The mean age of students in this study was 13.74 ± 1.66 years, with a range of 10 to 17 years. Out of 1330, 619 (47.0%) students were of normal BMI and 479 (36.0%) students were underweight. The prevalence of overweight and obesity were 145 (11.0%) and 87 (6.0%) among students respectively (Figure 1).

The role of various dietary factors in obesity was assessed. Type of diet, consumption of junk food, and carbonated drinks were associated with obesity. One hundred fifty-one (19.4%) students who were obese had mixed type of diet. Two hundred ten (18.5%) obese students consumed junk food. Seventeen (29.3%) obese students consumed more than four times per week. One hundred eighty-three (19.3%) obese students consumed carbonated drinks. Sixteen (39.0%) students consumed carbonated drinks more than four times per week. All these variables which were associated with obesity were found to be statistically significant (Table 1).

**Table 1:** Association of overweight / obesity with dietary variables of study participants * p less than 0.05 is found to be statistically significant.

Variables	Subtype	Non-overweight/ non-obese	Overweight/obese	Chi-square	P value
Type of diet	Vegetarian	470 (85.3%)	81 (14.7%)	4.915	0.027*
	Mixed diet	628 (80.6%)	151(19.4%)		
Consumption of Junk food	Yes	923 (81.5%)	210(18.5%)	6.325	0.012*
	No	175 (88.8%)	22 (11.2%)		
Frequency per week	Once and less than 1	521 (87.6%)	74 (12.4%)	44.183	0.000*
	Twice	254 (77.7%)	73 (22.3%)		
	Thrice	72 (68.6%)	33 (31.4%)		
	Four times	35 (72.9%)	13 (27.1%)		
	>4 times	41 (70.7%)	17 (29.3%)		
Carbonated drinks	Yes	764 (80.7%)	183(19.3%)	8.076	0.004*
	No	334 (87.2%)	49 (12.8%)		
Frequency per week	Once and less than 1	476 (86.5%)	74 (13.5%)	45.893	0.000*
	Twice	179 (72.8%)	67 (27.2%)		
	Thrice	64 (79.0%)	17 (21.0%)		
	Four times	20 (69.0%)	9 (31.0%)		
	>4 times	25 (61.0%)	16 (39.0%)		

Table 2 shows association of obesity with duration of hours of sleep. It was found that 167 (19.1%) students who were obese slept for six to eight hours of duration. However it was not found to be statistically significant.

**Table 2:** Prevalence of overweight / obesity according to hours of sleep

Hours of Sleep	Non-overweight/ non-obese	Overweight/obese	Total	Exact test	P value
<6 hours	14 (87.5%)	2 (12.5%)	16	4.679	0.189
6-8 hours	707 (80.9%)	167(19.1%)	874		
8-10 hours	353 (85.7%)	59 (14.3%)	412		
>10 hours	24 (85.7%)	4 (14.3%)	28		
Total	1098	232	1330		

Table 3 shows 64 (22.9%) of the children who were obese or overweight belonged to 14 years of age. We could also find the number of children (eight, 8%) who were non-obese were the least in the 17 years of age group. 

**Table 3:** Prevalence of overweight / obesity according to age

Age (years)	Non-overweight/ non-obese		Overweight/ obese		Total	Chi-square	P value
10	23	95.8%	1	4.2%	24	13.025	0.072
11	147	87.0%	22	13.0%	169		
12	118	86.1%	19	13.9%	137		
13	156	82.8%	32	17.2%	188		
14	216	77.1%	64	22.9%	280		
15	288	81.4%	66	18.6%	354		
16	142	84.5%	26	15.5%	168		
17	8	80.0%	2	20.0%	10		
Total	1098	100%	232	100%	1330		

Table 4 shows the association of obesity with variables like gender, type of school, religion, socio-economic class, type of family and family history of obesity. One hundred sixty-two (20.2%) students who were from private schools were more obese than those who studied in government schools. This also suggests people who belonged to better socio-economic class were obese (80, 16.6%). Family history of obesity increased the students to develop obesity at such a young age. However it was found to be statistically significant with type of school, socio-economic class, and family history of obesity.

**Table 4:** Association of overweight and obesity with socioeconomic factors of study participants * p less than 0.05 is considered statistically significant

Variables	Subtype	Non- overweight/ non-obese	Overweight/ obese	Chi-square	P value
Gender	Boys	562 (82.0%)	123 (18.0%)	0.258	0.612
	Girls	536 (83.1%)	109 (16.9)		
Type of school	Government school	460 (86.8%)	70 (13.2%)	10.979	0.001*
	Private school	638 (79.8%)	162 (20.2%)		
Religion	Hindu	957 (82.8%)	199 (17.2%)	0.833	0.659
	Muslim	127 (80.4%)	31 (19.6%)		
	Others	14 (87.5%)	2 (12.5%)		
Socio-economic class	Class 1	58 (72.5%)	22 (27.5%)	18.321	0.001*
	Class 2	219 (78.8%)	59 (21.2%)		
	Class 3	309 (83.1%)	63 (16.9%)		
	Class 4	400 (83.3%)	80 (16.6%)		
	Class 5	112 (93.3%)	8 (6.6%)		
Type of family	Nuclear	698 (82.2%)	151 (17.8%)	0.226	0.893
	Joint	271 (83.4%)	54 (16.6%)		
	Three generation	129 (82.7%)	27 (17.3%)		
Family history of obesity	Present in Father only	70 (61.4%)	44 (38.6%)	102.281	0.00*
	Present in Mother only	90 (65.7%)	47 (34.3%)		
	Present in Both father & mother	20 (55.6%)	16 (44.4%)		
	Absent	918 (88.0%)	125 (12.0%)		

Table 5 shows association of obesity with sedentary behavior. Increased screen time and decreased physical activity are the contributing factors to the epidemic of obesity in children. Fourteen (36.8%) students who watched TV, 26 (21.8%) students who used mobile phones and nine (29.0%) students who played video games for more than 10 hours per week had obesity. One hundred forty (22.0%) students who opted for motorized vehicles over walking and cycling as a mode of transport were obese. These variables were found to be statistically significant.

**Table 5:** Association of overweight / obesity with sedentary behavior * p less than 0.05 is found to be statistically significant

Variables	Sub type	Non- overweight/ non-obese	Overweight/ obese	Chi-square	P value
Duration of Watching TV (Total Hours per week)	<5 hours	377 (84.5%)	69 (15.5%)	14.415	0.006*
	5-10 hours	378 (83.4%)	75 (16.6%)		
	10-15 hours	189 (78.8%)	51 (21.2%)		
	>15 hours	24 (63.2%)	14 (36.8%)		
Mobile phone and internet (Total hours per week_	<5	420 (82.2%)	91 (17.8%)	10.214	0.017*
	5-10	225 (78.7%)	61 (21.3%)		
	>10	93 (78.2%)	26 (21.8%)		
Video game playing, Total hours per week	<5	274 (81.3%)	63 (18.7%)	7.835	0.050*
	5-10	101 (77.1%)	30 (22.9%)		
	>10	22 (71.0%)	9 (29.0%)		
Mode of transport	Using Motorized vehicle	498 (78.0%)	140(22.0%)	17.242	0.000*
	Walking and bicycle	600 (86.7%)	92 (13.3%)		

## Discussion

A systematic review done by Ranjani et al. [1] found that the prevalence of overweight and obesity in children ranged from 3% to 24.7% and 1.5% to 28% respectively in India. A study conducted by Jain et al. [8] in 2018 in Meerut, Uttar Pradesh, among adolescents age group of 10-19 years found the prevalence of overweight and obese to be 17.4% and 6.9%. A study done by Vedavathy et al. [9] showed the prevalence of obesity in the age group of 11-19 years to be 5.9%. A study found the prevalence of overweight and obesity to be 18.7% and 5.8% respectively [10]. This depicts the rise of childhood obesity.

The maximum prevalence (22.9%) of obesity in children was reported at the age of 14 years but the association between obesity and age was not statistically significant (p value = 0.072) (Table 3). Similar results were seen in a study by Booth et al. [11] done in Australia revealed that there was no consistent relationship between overweight and obesity and age. During periods of rapid development up to the age of 15, the number of fat cells increases, after which increased fat ordinarily accumulates by increasing the size of the fat cells already present. In a study done by Elangovan proportion of overweight among adolescents was higher in pubertal age group at 13-15 years of life [12].

In this study, boys (18.0%) were more overweight and obese as compared to girls (16.9%) but, the difference was not found to be statistically significant (Table 3). The reason for higher overweight/obesity in boys may be the cultural benefits that boys enjoy in India. They get more quantity of food at home, more snacks as they have greater freedom to go out and they also participate negligibly in household work. In contrast to the present study, a study done in Ludhiana in 2014 by Chhatwal et al. found that no major gender difference in obesity prevalence was seen among children [13]. A study done in north Chennai in 2018 by Elangovan observed a statistically significant higher proportion of overweight and obesity among girls (23.9%) as compared to boys (20.1%) [12]. The present study shows that the prevalence of overweight/obesity was significantly higher in those students who had a family history of obesity (Table 4). Type of diet, consumption of junk food and carbonated drinks were associated with obesity (Table 1). One hundred fifty-one (19.4%) students who were obese had mixed type of diet. Two hundred ten (18.5%) obese students consumed junk food. Seventeen (29.3%) obese students consumed more than four times per week. One hundred eighty-three (19.3%) obese students consumed carbonated drinks. Sixteen (39.0%) students consumed carbonated drinks more than four times per week. All these variables which were associated with obesity were found to be statistically significant. Highest prevalence of overweight/obesity was found in students (12.0%) who had a history of obesity in both father and mother. This could be due to genetic factors, lifestyle habits and diet trends that were followed in households, which are important etiological variables in the prevalence of obesity/overweight. In a study done in Italy in 2018 by Corica et al., childhood obesity was positively associated with family history of obesity [14]. 

A higher prevalence of overweight/obesity was found in those students who had consumed junk food three or more times per week and it was statistically significant. A study done by Patil observed that children who had food more than four times per week outside in a restaurant had a higher risk of obesity [15]. A study done in north Chennai in 2018 by Elangovan observed that higher prevalence of overweight and obesity was found in those who like junk food as compared to those who avoid junk food and the difference was found to be statistically significant [12]. A significantly higher prevalence of overweight/obesity was found in those students who had carbonated drinks four or more times per week. A study found a statistically significant association between daily soft drink consumption and overweight and obesity, with an odds ratio of 1.14 among school-going adolescents [16].

Table 5 shows that the highest prevalence of overweight/obesity was seen in students (30.6%) who participate in outdoor games once a week and the least is seen in those students (20.4%) who participated in outdoor games daily and the difference in prevalence of overweight/obesity was statistically significant with respect to frequency of outdoor games per week. A study done by Gebremichael et al. in Ethiopia in 2015 shows that there was an inverse relationship between overweight/obesity and physical activity (more than two times/week) [17]. In a study done by Navti et al., moderate (more than two to four times/week) and high (more than four to seven times/week) physical activities were significantly associated with a lower prevalence of overweight/obesity [18]. The finding of this study was similar to other studies where a positive association was found that the odds of becoming obese were less when exercising regularly [17]. A study done in north Chennai in 2018 by Elangovan observed that the proportion of overweight was significantly lower among the adolescents who participated in outdoor games for more than six hours in a week (7.3%) than those who did not participate (38.6%) [12]. A study done by Sharma shows that incidence of obesity and overweight was found much higher among adolescents participating less than two hours/week in physical activity [19]. 

In the present study, 17.6% of students were overweight/obese who participated in household activities and 16.8% of students were overweight/obese who did not participate in household activities. A study by Elangovan observed that obesity was significantly higher among the children who did not perform any household chores (49.3%) than among those who performed them (13.9%) [12].

As seen in Table 5, a high prevalence of overweight/obesity was seen in those students (36.8%) who watched television >15 hours a week and it was statistically significant. This could be due to increase in screen time leading to less involvement in physical activity, and trend of consuming food while watching television and getting influenced by advertisement of fast food which make them curious to consume junk and processed food. A study done in south India in 2018 by Doss shows that the prevalence of overweight and obesity was significantly high in those students who watched television for one to two hours per day [10]. The National Institute of Health, US also considers television watching more than two hours/day as a significant risk factor for obesity [20].

The present study concluded that a statistically significant difference in the prevalence of overweight/obesity was found between those students who played video games as compared to those who did not play video games (Table 5). Higher prevalence of overweight/obese was seen in students who spent >10 hours per week playing video games. Falbe et al.'s study found that video game/computer game hours were associated with larger BMI gains in children [21]. A study done in Northeast India in 2016 by Bhattacharya observed that higher prevalence of obesity in those children who play video or computer games more than three to four hours per day [22]. Higher prevalence of overweight/obese was seen in students who spent >10 hours per week playing video games. A study done by Haghjoo et al. found that adolescents who use more screen time were 1.27 times more likely to develop overweight/obesity [23].

A statistically significant difference in prevalence of overweight/obesity was found between those students (22.0%) who used motorized vehicles, as compared to those (13.3%) who used walking and bicycle. It may be because of use of transport contributes to sedentary activity by reducing the cost of energy. Elangovan observed that the proportion of overweight among those come by car, bus, and motorcycle was 31.0%, which was significantly higher than those who went to school by bicycle and walking (6.1%) [12]. It may be because of use of transport contributes to sedentary activity by reducing the cost of energy.

The prevalence of overweight/obesity was found more in non-vegetarian students (19.4%), as compared to vegetarian students (14.7%) with p = 0.027 (Table 4). In a study done by Thaddanee et al., the prevalence of obesity and overweight was significantly higher among those who took a mixed diet as compared to vegetarians [24].

Regular health education on nutrition should be given to all children in schools. It should include healthy food habits, nutritive values of food items, lifestyle and behavioral modification. Limitations of this study are that the measurement of a child’s dietary habits and level of physical activity may have recall bias. As the information on parental education was obtained by the students, the information may not be fully correct. The secondary sexual characteristics changes were not taken into consideration while assessing the obesity.

## Conclusions

To address the increase in childhood obesity, both parents and children should be educated on the risks of obesity. The government should implement and enforce legislation targeting artificially sweetened soft drinks, nutritional labeling, and restrictions on advertisements, while also enhancing programs focused on childhood nutrition and lifestyle. The Government of India’s National Program on Prevention and Control of Non-Communicable Diseases has a school component that requires further development. Additionally, raising awareness among all stakeholders through education and motivation is crucial. This approach will significantly contribute to preventing childhood obesity and curbing the rise of non-communicable diseases like diabetes and cardiovascular disease in India.

## Appendices

**Table 6:** Proforma -Prevalence of obesity and associated factors in school going children of North Karnataka city

Prevalence of obesity and associated factors in school going children of North Karnataka city	
Name	
Age	
Gender	
Class	
Religion	
Address	
Father’s qualification	
Father’s occupation	
Mother’s qualification	
Mother’s occupation, family monthly Income, socio-economic class	
Type of family:	
History of any chronic illness	
History of hospitalizaton in past 6 months	
History of any endocrinal disorders	
History of any drug intake	
History of familial obesity: yes/no, Physical activity assessment - Participation in outdoor games, indoor games, household activities, walking, Time spent on watching TV, internet and mobiles, video games	
Mode of transport to school: Motorised vehicle / walking or bicycle	
Hours of sleep:	
Type of diet: vegetarian / mixed	
Consumption of junk food- Yes or No If yes then Frequency……………….	
Consumption of carbonated drinks – If yes then Frequency……………….	
Weight in Kgs	
Height in cms	
Waist circumference in cms	
Hip circumference in cms	
BMI	
